# Knowledge, attitude, and practice of insulin among diabetic patients and pharmacists in Egypt: “cross-sectional observational study”

**DOI:** 10.1186/s12909-024-05367-5

**Published:** 2024-04-09

**Authors:** Alaa H. Mohamed, Maggie M. Abbassi, Nirmeen A. Sabry

**Affiliations:** Department of Clinical Pharmacy, Faculty of Pharmacy, Cairo University, Kasr El-Aini St, P.O. Box: 11562, Cairo, Egypt

**Keywords:** Knowledge, Attitude, Practice, Insulin, Self-confidence, Pharmacists, Diabetic patient, Egypt

## Abstract

**Background:**

Diabetes recently has been identified as a growing epidemic. Although insulin’s vital role in both types of diabetes, it is considered one of the harmful medications if used incorrectly. In Egypt, effective usage of insulin remains a challenge due to insufficient knowledge of insulin and diabetes management, leading to errors in insulin therapy. As pharmacists are experts in pharmacological knowledge, they are uniquely situated to assess adherence to treatment regimens, the effect of drug therapy, or potential alterations in drug therapy to meet patient goals. To provide effective patient education and counseling, community pharmacists in Egypt should be efficiently knowledgeable about diabetes and insulin.

**Objective:**

To identify the knowledge, attitude, and practice of pharmacists and patients about insulin. To identify pharmacists’ educational preparedness and confidence in counseling diabetic patients.

**Methods:**

A descriptive, cross-sectional study was conducted with two knowledge, attitude, and practice surveys. This study was carried out from September 2016 to February 2023. Face-to-face interviews were conducted with patients, and a paper-based questionnaire was administered to pharmacists. The two questionnaires were adapted from previous studies.

**Results:**

A total of 492 patients and 465 pharmacists participated in this study. The mean knowledge score of correct answers among patients and pharmacists was 10.67 ± 1.9 and 15 ± 3.6. Most of the patients and pharmacists had a positive attitude regarding insulin’s role in improving health and to better control blood glucose. On the negative side, around half of the patients reported that they believe that regular use of insulin leads to addiction, while only 14.5% of the pharmacists believed that insulin could cause addiction. Self-confidence scores for pharmacists differed statistically with sex, years of experience, and pharmacist’s direct exposure to diabetic patients.

**Conclusions:**

This study uncovers considerable deficiencies in patients’ and pharmacists’ knowledge about insulin therapy. This study also strongly recommends higher education and a more structured pharmacist training schedule.

**Supplementary Information:**

The online version contains supplementary material available at 10.1186/s12909-024-05367-5.

## Introduction

Diabetes mellitus (DM) is a heterogeneous disorder of metabolism characterized by chronic high blood glucose levels resulting from either deficiency in insulin secretion or insensitivity of tissues to insulin or both [1]. Diabetes recently has been identified as a growing epidemic due to its significant increase over the past 10 years. Egypt is listed as the 9th among the top 10 countries with diabetes, and the number of adult diabetic patients was 8,850,400 in early 2020, predicted to double to 16.9 million by 2045 [2]. Although insulin plays a vital role in both types of DM, it is considered one of the most harmful medications if used incorrectly. For this reason, it is recognized as one of the five “high alert” medications that have the highest hazard of inducing harm to patients due to medication errors [3].

In developing countries, such as Egypt, the effective use of insulin in the management of DM remains a challenge due to several factors. An important one is insufficient knowledge of insulin and diabetes management on the part of healthcare providers and patients, leading to errors in insulin therapy [4]. Consequently, preventable, and life-threatening complications, such as hyperglycemia and hypoglycemia, may occur [5]. El-Khawaga et al. [6] and Soliman et al., [7] reported that among the most common insulin misuses in Egyptian diabetic patients are managing hypoglycemic symptoms and the proper way of mixing insulin, where only 24.1% knew the conditions leading to hypoglycemia and only 13.0% knew the proper way of mixing insulin, disposal of the sharp syringes, needles, and lancets. Another study [8] identified some major errors in the self-administration of insulin among diabetic patients such as not removing air bubbles from the syringe before injecting, injection on the scar leading to lipodystrophy, not rotating the injection site, using insulin while it is cold that can result in increasing pain during injection, and unsafe disposal of used needles or syringes. Therefore, the assessment of diabetes knowledge, attitude, and practice (KAP) has become essential for the development of control programs and techniques for effective health education and patient counseling. This aids in ensuring that each diabetic patient has satisfactory information and is interested in leading a better life [9]. Various KAP studies have been conducted worldwide on patients receiving insulin [10–13]. Various Egyptian studies have emphasized diabetes epidemiology [2, 14, 15], but no study was conducted in Egypt regarding KAP in diabetic patients on insulin.

Pharmacists have a potential role in identifying and preventing medication misuse among diabetic patients in Egypt [16]. As they are experts in pharmacological knowledge, pharmacists are uniquely situated to assess adherence to treatment regimens, the effect of drug therapy, or potential alterations in drug therapy to meet patient goals [17]. With prolonged working hours and commonly no appointment needed for service, resulting in easier accessibility and lower cost, therefore Egyptian patients seek immediate health advice from community pharmacists rather than physicians [18]. Pharmacists who have good knowledge and are well-educated could implement group education for diabetic patients, thus making the load on primary health care centers easier and giving participants the time they need. To provide effective patient education and counseling, community pharmacists in Egypt should be efficiently knowledgeable about diabetes and insulin. Therefore, there is a need to survey whether community pharmacists in Egypt possess adequate knowledge and attitudes to deliver proper patient education services.

## Methods

### Study aim

This study aims to assess the knowledge, attitude, and practice of pharmacists and patients about insulin, assess pharmacists’ educational preparedness and confidence in counseling diabetic patients, and measure patients’ satisfaction with their diabetes care.

### Study design

This descriptive cross-sectional study was conducted in Greater Cairo (Giza and Cairo), Egypt, using a KAP survey regarding insulin therapy among pharmacists and diabetic patients, between September 2016 and February 2023.

### Study population

#### Patients

The inclusion criteria for this study were diabetic patients > 18 years old, patients on insulin treatment, and those willing to participate in the study.

The exclusion criteria were the inability to be interviewed and patients taking only oral hypoglycemic drugs for diabetes mellitus.

#### Pharmacists

The inclusion criteria for this study were as follows:

Fully licensed pharmacists working at community or hospital pharmacies.

### Sample size

In Greater Cairo, it was estimated that there were 60,000 registered pharmacists and 63,000 licensed community pharmacies at the general pharmacy syndicate until 2016 [19]. Yellow pages were used to help the author determine the actual number of pharmacies in each district so that the author can be able to determine the percentage of pharmacists that should be recruited from each district in Cairo and Giza [20], and the number of pharmacies to be visited in each district was calculated by cross-multiplication of the sample size, the number of pharmacies in each district, and the total number of pharmacies. After calculating the number of pharmacies to be visited in each district, the researcher randomly visited the pharmacies. Another study performed in Egypt, in addition to the International Diabetes Federation reports were used to determine the approximate total number of diabetic patients in Egypt [14, 21].

The sample size was calculated using Raosoft sample size calculation software, in which the population size (the number of pharmacists was 60,000 and the number of diabetic patients was 8 million), response distribution was 50%, and margin of error and confidence interval were set at 5 and 95%, respectively [22]. A minimum sample size of 382 pharmacists and 385 patients was calculated.

### Study sites

Patients were recruited from public hospitals in great Cairo, which is affiliated with the Ministry of Health and Population and the Ministry of Higher Education.

### Ethics approval

The study protocol was approved by the Research and Ethics Committee for Experimental and Clinical Studies of the Faculty of Pharmacy, Cairo University. Approval has been valid since May 2016.

### Consent to participate

The Research Ethics Committee for Experimental and Clinical Studies at the Faculty of Pharmacy, Cairo University waived the requirement for the use of the standard written informed consent. Instead, written informed consent was replaced by verbal consent. Both questionnaires included an introductory cover letter asking candidates’ permission to participate after providing a brief description of the topic, the objectives of the research, and the duration needed to complete the questionnaire. Confidentiality of data was assured by the anonymity of the questionnaire. Acceptance of the interview was considered consent to participate in the study.

### Questionnaires development and validation

#### Preliminary questionnaire development

Questionnaires were used to evaluate pharmacists’ and patients’ knowledge, attitudes, and practices toward insulin use. The two questionnaires were adapted from previously published studies in English [5, 10, 23]. The final validated form of the pharmacists’ questionnaire was in English language where pharmacy education in Egypt is in English. The patients’ questionnaire was developed in English and translated into Arabic (the official language in Egypt) by a professional English/Arabic language translator.

#### Pilot testing and questionnaire validation

Content validity was tested by distributing the questionnaire to staff members of the Clinical Pharmacy Department at Cairo University. They were asked to complete the questionnaire and provide comments on both structure and content accuracy. Their comments were used to improve language and/or content. A few modifications were made.

Face validity was then assured by carrying out a pilot study on 50 pharmacists and 50 patients. Relevant modifications were instituted before the commencement of actual data collection. The data collected from the pilot study were not included in the results. Age question was changed to an open-ended question (continuous data), and the “do not know” option was added to both questionnaires. Regarding the pharmacists’ questionnaire, three open-ended questions were deleted to decrease the length and time needed to complete the questionnaire, and the “one or multiple answers” word was added to questions 18,19, 24, and 30. Four questions were deleted from the attitude section of the pharmacists’ questionnaire. Regarding the patients’ questionnaire, the attitude scale was changed from 5 points to a 3-point Likert-scale for ease of understanding the difference between scales in the patients’ category.

##### Reliability of patients’ questionnaire

The patient’s attitude scale was meant to be a formative construct, where each item contributes a unique aspect to the construct, and items do not necessarily correlate with each other. This is because each item represents a different dimension that forms or contributes to the overall construct, and changes in one item do not necessarily imply changes in the others. Indeed, the correlation between the attitude items did not exceed 0.25 for any pair of items.

This contrasts with reflective constructs, where all items are expected to correlate highly with each other as they reflect the same underlying construct. Indeed, the items included in the attitude scale were meant to assess the satisfaction of patients with the different aspects of insulin therapy such as cost, ease of use, associated pain, and perceived glycemic control. These items are not correlated with each other. Thus, calculating Cronbach’s alpha is not applicable in such a case. This contrasts with reflective constructs (e.g. experience with physician), where all items are expected to correlate highly with each other as they reflect the same underlying construct which permits the calculation of indices such as Cronbach’s alpha. For the “experience with the physician” which was a reflective construct. The reliability was 0.8 and 0.77 for the pilot and main studies, respectively.

##### Reliability of the pharmacists’ questionnaire

The attitude of the pharmacists towards was also regarded as a formative rather than a reflective measure which makes Cronbach’s alpha not suitable. Thus, reliability was only calculated for the confidence scale**,** which was 0.88 and 0.85, for the pilot and main studies, respectively.

#### The final form of the questionnaire

##### Patients’ questionnaire

The final patient questionnaire (Additional file 1) was divided into 3 parts. The first part (nine questions) covered demographics and other patient characteristics, including age, sex, occupation, education level, residence, monitoring blood glucose at home, method of insulin administration (needle/syringe or pen), and duration of insulin use. The second part was designed to assess the patients’ KAP regarding insulin. It consists of 36 questions grouped into three sections. Section I included 15 questions (10 (Yes/No) questions, 3 close-ended (multiple choice), and 2 open-ended) about knowledge regarding insulin, covering the timing of short insulin administration, insulin route of administration, mixing of insulin different types, injection process, and insulin side effects. Section II included 15 questions based on a Likert scale of three levels (disagree/neutral/agree) to evaluate the attitude of patients towards insulin (benefits, fears about side effects, effectiveness, compliance, and cost). Section III assessed the patients’ practice of insulin self-injection using a 6-step checklist. The third part was composed of three questions assessing patients’ satisfaction with diabetes care, based on a Likert scale of 3 levels and two questions about reasons for dissatisfaction.

##### Pharmacist’s questionnaire

This was composed of three parts. The first part (8 questions) covered the demographic data of pharmacists (age, sex, year of graduation, years of practice as a pharmacist, education level (postgraduate studies), graduating university, and direct exposure to patients on insulin during practice). The second part assessed pharmacists’ knowledge and attitudes regarding DM and insulin. It was composed of 38 questions, grouped into two sections. Section I (Knowledge) was composed of 27 questions (1 open-ended question, 15 Yes/No, and 11 multiple-choice questions) related to pharmacists’ knowledge about the nature of insulin, from where insulin is secreted, which patients require insulin, insulin storage, insulin use in pregnancy, switching between different types of insulin, side effects and contraindications of insulin, hypoglycemia, diabetic ketoacidosis, insulin injection sites, decreasing pain during injection, and preparation of insulin injection. Section II (11 questions) was designed to assess pharmacists’ attitudes towards insulin (benefits, side effects, effectiveness, compliance, cost), using a 5-point scale from strongly disagree to strongly agree. The third part (7 questions) covered questions about the pharmacists’ confidence in their therapeutic knowledge regarding insulin using a 5-point scale ranging from “poor” to “excellent” (Additional file 2).

#### Questionnaires distribution

##### Patients

A face-to-face interview was used with the patient’s category. The interview was conducted in a single session of 10–15 minutes and filled at once.

##### Pharmacists

For this sector, a paper-based questionnaire was distributed by the investigator to be self-filled by the participants in their pharmacies. The completion time of the survey was designed to be 12 minutes but, in some instances, it took more than that according to the pharmacist’s spare time. Some pharmacists asked to finish the survey in a few days to complete it in their spare time. Surveys returned entirely blank were counted as non-responders and were not used in the analysis.

### Scoring system

Regarding the knowledge section, a score of one was given for correct answers and a zero for incorrect, and “do not know” answers. The grading of participants’ knowledge according to their total score was as follows: adequate knowledge > 75%, moderately adequate knowledge 51–75%, and inadequate knowledge< 50% [24]. Regarding the attitude and practice sections, the data were expressed as frequencies and percentages. For the self-confidence part of the pharmacists’ questionnaire, scoring was performed as follows: a score from 1 to 5 was given to responses from poor to excellent (< 10 points for poor self-confidence, 10–20 points for good self-confidence, and > 20 points for excellent self-confidence).

### Statistical analysis

Statistical analyses were performed using Statistical Packages for Social Science (SPSS) version 23. The figures were produced using Microsoft Excel 2010. Nonparametric tests (Mann–Whitney U test and Kruskal-Wallis test) were performed to relate knowledge scores and demographic variables. Statistical significance was set at *P* < 0.05. The data collected using the preliminary questionnaire were not used in the final analysis.

## Results

### Patients

A total of 492 patients were included in the study, of whom 369(75%) were female with an age range of 51–60 years (175(35.6%)). More than half of the respondents, 277(56.3%) were uneducated. Unemployed patients, 363(73.8%) were the highest proportion. Patients’ demographics are shown in Table 1.

**Table 1:** Patients’ characteristics presented as frequency and percentage (*N* = 492)

Variable		Frequency (%)	Total N (%)
Sex	Male	123(25%)	492(100%)
Age	18–30	37(7.5%)	492(100%)
	31–40	43(8.7%)	
	41–50	108(22%)	
	51–60	175(35.6%)	
	61–70	107(21.7%)	
	71–80	21(4.3%)	
	> 80	1(0.2%)	
Education	Primary	57(11.6%)	483(98.2%)
	Secondary	108(22%)	
	Higher education	41(8.3%)	
	Uneducated	277(56.3%)	
Employment	Unemployed	363(73.8%)	490(99.9%)
	Self-employed	78(15.9%)	
	Retired	45(9.1%)	
	Student	4(0.8%)	
Residence	Rural area	75(15.2%)	489(99.4%)
	Urban area	414(84.1%)	
Glucose home monitoring by glucometer	Yes	188(38.2%)	492(100%)
If yes, who trained you to use the device	Doctor	70(37.2%)	
	Pharmacist	41(21.8%)	188(38.2%)
	Nurse	9(4.8%)	
	Relatives	43(22.8%)	
	Myself	17(9%)	
	Other patients	8(4.2%)	

Most patients 325(66.1%) used syringes, and 164(33.3%) used pen devices to administer insulin. Most of the respondents (281(57.1%)) had been on insulin treatment for a period between 1 and 10 years, 76(15.2%) had used insulin for less than 1 year, and 132(26.8%) had used insulin for more than 10 years.

#### Knowledge, attitude, and practice of patients towards insulin

Of the 15 questions related to insulin knowledge, the mean score of correct answers was 10.67 ± 1.9, compared to the maximum score of 16, and 458(93.1%) showed moderately adequate knowledge. The frequency distribution of the responses regarding knowledge is presented in Table 2.

**Table 2:** A summary of responses to questions about knowledge of patients towards insulin use represented as frequency and percentage (*N* = 492)

Item	Correct answer	Frequency (%)
Short-acting insulin should be taken	Before meals	467(94.9%)
Insulin should be given	Sc	366(74.4%)
If two types of insulin are to be mixed, is/are there any precautions (s) you should take?	Gentle mixing, or rotating	179(36.4%) 63(12.8%)
Abdomen is a site of insulin injection	Yes	457(92.9%)
The thigh is a site of insulin injection	Yes	475(96.5%)
The upper arm is a site of insulin injection.	Yes	479(97.4%)
Insulin should be injected while it is cold.	No	166(33.7%)
To decrease pain, you should use a thick needle.	No	445(90.4%)
You should remove air bubbles.	Yes	395(80.3%)
The angle to administer insulin injection is 45 degrees.	No	325(66.1%)
You should rotate the injection site.	Yes	462(93.8%)
Insulin should be stored in	Fridge	461(93.7%)
The syringe should be used for one time only.	No	418(85%)
Do you know the side effects of insulin?	Yes	39(7.9%)
If yes, what Side effect/s of insulin is/are	Allergy Weight gain Hypoglycemia	3(0.6%) 8(1.6%) 5(1%)

*Sc stands for subcutaneous

The findings of this study revealed that knowledge differed significantly with sex (*p* = 0.002), and female patients were more knowledgeable. In addition, knowledge differed with the duration of insulin use (*p* = 0.021), with those who had been using insulin for > 30 years having the highest scores, while those who had an insulin treatment history of less than 6 months scored the lowest. Knowledge of insulin use was not associated with age, education, employment, or residence (Table 3).

**Table 3:** Predictor variables of knowledge about insulin among patients (*N* = 492)

Variable	Knowledge score	
	Median	***P*** value
*Sex*		0.002*
Male	10	
Female	11	
*Age*		0.320
18–30	10	
31–40	10	
41–50	11	
51–60	10	
61–70	11	
71–80	11	
*Education*		0.477
Primary	11	
Secondary	10	
Higher education	10	
Uneducated	11	
*Employment*		0.324
Unemployed	10	
Self-employed	10	
Retired	10	
Student	9	
*Residence*		0.558
Rural area	11	
Urban area	11	
*Duration of insulin use*		0.030*
< 6 months	10	
6–12 months	11	
> 1 year-10 years	10	
> 10 years–20 years	11	
> 20 years–30 years	11	
> 30 years	12	

*Kruskall Wallis test is used

**Fig. 1:**
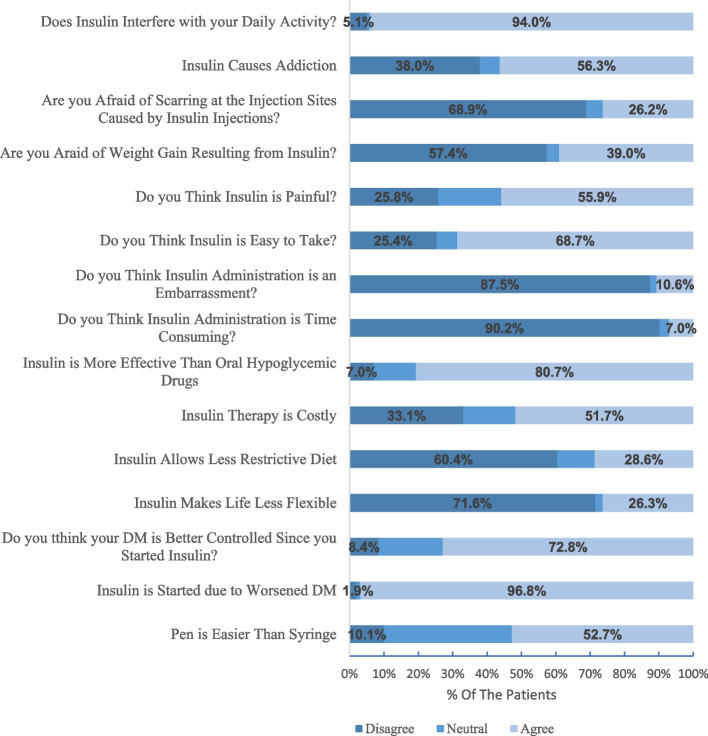
Perception of patients towards insulin use and administration

Approximately half of the patients (251(52.7%)) thought that the pen device would be easier to use, while 48(10.1%) did not agree. Most patients, 461(96.8%) perceived that insulin was initiated due to worsened DM. A summary of patients’ attitudes and perceptions is presented in Fig. 1.

The differences in the perceptions of patients regarding insulin regimen stratified by age, sex, education status, employment, and method of insulin administration are shown in Table 4.

**Table 4:** Differences in the attitude of patients towards insulin use stratified by age, gender, education status, employment, and insulin administration type (N = 492)

Variable	Age group								Gender			Education					Employment					Insulin administration device		
	Median							***P*** value	Median		***P*** value	Median				***P*** value	Median				***P*** value	Median		***P*** value
	18–30	31–40	41–50	51–60	61–70	71–80	> 80		male	female		primary	secondary	higher education	uneducated		unemployed	self-employed	retired	student		pen	needle/syringe	
Do you think insulin administration with a pen is easier than syringes?	3	2	3	3	2	3	2	0.067	3	3	0.429	3	3	3	2	0.046*	3	3	3	2	0.194	3	2	0.000*
Insulin started due to worsened DM	3	3	3	3	3	3	3	0.849	3	3	0.886	3	3	3	3	0.450	3	3	3	3	0.393	3	3	0.269
Do you think your DM has been better controlled since you started insulin?	3	3	3	3	3	3	3	0.134	3	3	0.054	3	3	3	3	0.142	3	3	3	3	0.174	3	3	0.226
Insulin makes life less flexible	2	1	1	1	1	1	1	0.000*	1	1	0.871	1	1	1	1	0.131	1	1	1	1	0.088	1	1	0.814
Insulin allows a less restrictive diet	1	1	1	1	1	1	1	0.725	1	1	0.831	1	1	1	1	0.295	1	2	1	1	0.138	1	1	0.094
Insulin therapy is costly	3	3	3	3	3	2.5	1	0.107	2	3	0.232	2	3	3	2.5	0.089	3	2	2	3	0.872	3	3	0.280
Insulin is more effective than oral hypoglycemic drugs	3	3	3	3	3	3	3	0.008*	3	3	0.930	3	3	3	3	0.000*	3	3	3	2	0.005*	3	3	0.314
Do you think insulin administration is time-consuming?	1	1	1	1	1	1	1	0.602	1	1	0.007*	1	1	1	1	0.914	1	1	1	1	0.000*	1	1	0.287
Do you think insulin administration is an embarrassment?	3	3	3	3	3	3	1	0.057	1	1	0.838	1	1	1	1	0.907	1	1	1	1	0.186	1	1	0.930
Do you think insulin is easy to take?	3	3	3	3	3	3	1	0.144	3	3	0.331	3	3	3	3	0.145	3	3	3	3	0.999	3	3	0.704
Do you think insulin is painful?	2	3	3	3	2	3	1	0.001*	2	3	0.000*	3	3	2	3	0.081	3	3	2	2	0.289	3	3	0.415
Are you fearful of weight gain resulting from insulin?	1	2	3	1	1	1	1	0.015*	1	1	0.008*	2	1	1	1	0.722	1	1	1	1	0.041*	1	1	0.928
Are you afraid of scarring at the injection sites caused by insulin injections?	1	1	1	1	1	1	1	0.034*	1	1	0.001*	1	1	1	1	0.203	1	1	1	1	0.203	1	1	0.072
Insulin causes addiction	3	2	3	3	3	2	1	0.196	3	3	0.288	3	3	3	3	0.494	3	2	2.5	3	0.472	3	3	0.517
Does insulin interfere with your daily activity?	3	3	3	3	3	3	3	0.764	3	3	0.606	3	3	3	3	0.218	3	3	3	3	0.269	3	3	0.183

*Significance difference, Kruskall wallis test is used

There were 260(52.8%) patients who had good insulin administration practice. The mean score of correct practice was 2.61 ± 1.1 compared to the maximum score of 5 (Table 5).

**Table 5:** Frequency distribution of correct steps for insulin injection use (*N* = 492)

Steps of insulin injection	% of cases
Wash your hands well.	30.08%
Gently pinch a two- to three-inch fold of skin on either side of the cleaned injection site.	65%
Insert the needle into the skin.	89.22%
Leave the skin and leave the syringe in place for 5 seconds after injecting.	39.63%
Pull the needle out and press on the skin for 5 seconds.	39.2%
Check for bleeding.	2.2%

When asking patients if they were satisfied with their doctors, most of the patients, 304(61.8%), were satisfied while 115(23.4%) patients were not satisfied with diabetic care. The satisfaction of patients with physician-patient interactions is shown in Table 6. Of the 115 dissatisfied patients, 88 preferred to visit another diabetes clinic.

**Table 6:** Satisfaction of patients with physician-patient interaction (*N* = 492)

Experience of patients with Physician	Poor (%)	Good (%)	Excellent (%)	Total (%)
Feel free to talk to your physician	3(0.6%)	8(1.6%)	293(59.6%)	304(61.8%)
Enough time for consultation	1(0.2%)	20(4.1%)	283(57.5%)	304(61.8%)
Ease of contacting your physician	8(1.6%)	13(2.6%)	283(57.5%)	304(61.8%)

When asking patients about the causes of dissatisfaction with their physicians, 109(35.9%) and 107(35.2%) were dissatisfied because there was not enough time for consultation and no follow-up, respectively.

Of the 10 hospitals visited, 7 offered patient education sessions for their patients. The present study showed a statistical difference between the knowledge scores of patients recruited from hospitals that offered educational courses and those recruited from hospitals that did not (*P* = 0.015). Mann-Whitney U test was used, as shown in Table 7.

**Table 7:** The difference in knowledge scores between patients based on recruiting hospital (offered education courses or not) (*N* = 492)

Variable	Knowledge score		
	N (%)	Median	***P*** value
Patients recruited from hospitals offering patient education courses	358(72.8%)	10	0.015*
Patients recruited from hospitals not offering patient education courses	134(27.2%)	11	

### Pharmacists

An analysis of the sample demographics and descriptive statistics is presented in Table 8. Most respondents were females, 279(60.1%). The mean age was 28.4 ± 7.7. Only 62(13.3%) pharmacists held postgraduate degrees.

**Table 8:** Pharmacists’ characteristics represented as frequency and percentage (*N* = 465)

Variable		Frequency (%)	Total N (%)
Sex	Male	185(39.8%)	464(99.8%)
Age range	22–30 years 31–40 years 41–50 years 51–60 years > 70 years	302(64.9%) 90(19.4%) 20(4.3%) 9(1.9%) 1(0.2%)	422(90.8%)
Graduating university	Private Public	99(21.3%) 322(69.2%)	421(90.5%)
Postgraduate studies	Yes No	62(13.3%) 393(84.5%)	455(97.8%)
Type of postgraduate studies	Master PhD Pharm D Diploma	17(3.7%) 2(0.4%) 5(1.1%) 38(8.2%)	62(13.3%)
Years of experience as a pharmacist	< 1 year 1–5 years 6–10 years > 10 years	99(21.3%) 167(35.9%) 58(12.5%) 122(26.2%)	446(95.9%)
Exposure to patients	Yes No	329(70.8%) 110(23.7%)	439(94.4%)
How comfortable do you feel when managing a diabetic patient?	Very comfortable Somewhat comfortable Somewhat uncomfortable Very uncomfortable	234(50.3%) 141(30.3%) 71(15.3%) 10(2.2%)	456(98.1%)

### Knowledge and attitude of pharmacists towards insulin

The knowledge assessment revealed that 97(21%) of the pharmacists had inadequate knowledge,309(66.5%) had moderately adequate knowledge, and only 59(12.7%) had adequate knowledge. The overall mean score percentage of knowledge was 61.22 ± 12.9%. The response distribution to the questions about pharmacists’ knowledge regarding insulin use is shown in Table 9.

**Table 9:** Response distribution to questions about knowledge of pharmacists regarding insulin use represented as frequency and percentage (*N* = 465)

Item	Pharmacist’s correct answer	Frequency (%)
Insulin is	Hormone	368(79.1%)
Insulin is secreted by the pancreas by	Beta cell	334(71.8%)
Which patient requires insulin?	Type 1&Type 2 DM	216(46.5%)
Insulin can be injected into the abdomen?	Yes	386(83%)
Insulin can be injected into the gluteus?	Yes	89(19.1%)
Insulin can be injected into the deltoid?	Yes	262(56.3%)
Insulin can be injected into the thigh?	Yes	357(76.8%)
The distance to rotate on the same site is one thumb	Yes	276(59.4%)
Unopened insulin vials should be stored in *One or multiple answers	Refrigerator	431(92.7%)
Used insulin vials should be stored in *One or multiple answers	Room temperature Refrigerator	100(21.5%) 375(80.6%)
If a fridge is not available insulin vial could be stored in a clay pot containing water	Yes	303(65.2%)
The insulin pen has a needle of 31-gauge	Yes	71(15.3%)
Women who get pregnant should stop insulin	No	364(78.3%)
How should short-acting insulin be taken concerning meals?	Immediately before or directly after	337(72.5%)
Insulin should be injected while it is cold	No	259(55.7%)
To minimize pain associated with insulin injections, a 29-gauge needle should be used	No	71(15.3%)
Removing air bubbles from the insulin syringe before injecting	Yes	329(70.8%)
If two types of insulin to be mixed, is/are there any precautions (s) you should take concerning the vial?	Gentle mixing or rotating	148(31.8%)
Hands should be washed with soap and water before handling injection devices	Yes	402(86.5%)
The insulin vial should be kept at room temperature at least for 15 minutes before giving the injection	Yes	280(60.2%)
Air should be injected into the insulin vial before drawing insulin out of the vial	Yes	119(25.9%)
If drawing both soluble and isophane insulin into the same syringe the isophane insulin should be drawn into the vial first	No	118(25.4%)
Which of the following is/are the side effect/s of insulin? *One or multiple answers	Weight gain Hypoglycemia Allergy	233(40.1%) 177(30.5%) 47(8.1%)
Diabetic ketoacidosis can be developed in	Type 1 and Type 2 DM	185(39.8%)
Hypoglycemia is a blood glucose level	< 50 mg/dl < 70 mg/dl	208(44.7%) 171(36.8%)
What are the signs of hypoglycemia	All the above	356(76.6%)
What are the contraindications for insulin?	Allergy Hypoglycemia	58(12.5%) 47(10.1%)

When pharmacists were asked about the most preferred method for continued learning about DM and insulin, 180(41.5%) pharmacists considered online courses to be the most preferred method, 135(31.1%) preferred workshops, and 197(45.4%) considered brochures distributed to the pharmacy as a good way to continue learning about diabetes. Other options mentioned by 29(6.7%) pharmacists were campaigns, lectures, and short message services on their mobile phones, while the remaining pharmacists were not interested in continuing to learn about DM and insulin.

**Fig. 2:**
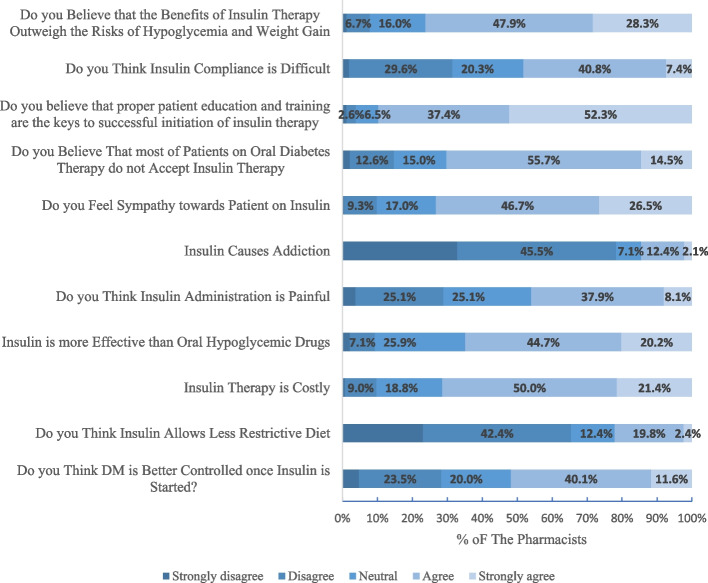
Pharmacists’ attitudes towards insulin use

Two out of every five pharmacists (40%) agreed that DM was better controlled once insulin was started, and 188(44.7%) thought that insulin was more effective than oral hypoglycemic drugs. In contrast, 210(50%) pharmacists agreed that insulin therapy is costly and 160(38%) thought that insulin is painful. A summary of pharmacist’s attitudes is shown in Fig. 2**.**

Differences in perceptions of pharmacists regarding insulin regimen stratified by age, gender, postgraduate studies, type of postgraduate studies, and years of practice as a pharmacist are shown in Table 10.

**Table 10:** Differences in perceptions of pharmacists towards insulin stratified by age, gender, postgraduate studies, type of postgraduate study, and years of practice (*N* = 465)

Variable	Age group							Gender			Postgraduate studies			Type of postgraduate study					Years of practice as a pharmacist				
	Median						***P*** value	Median		***P*** value	Median		***P*** value	Median				***P*** value	Median				***P*** value
	22–30	31–40	41–50	51–60	> 70			male	female		yes	No		Master degree	PhD	Pharm D	Diploma		< 1 years	1—5 years	6–10 years	> 10 years	
Do you think DM is better controlled once insulin is started?	3	4	4	3	2		0.419	4	4	0.851	3	4	0.002*	3	3	4	3	0.003*	3	3	4	3	0.018*
Do you think Insulin allows a less restrictive diet?	3	3	3	3	3		0.478	2	2	0.404	2	2	0.660	2	2	3	2	0.568	2	2	2	2	0.308
Insulin therapy is costly?	3	3	3	3	3		0.255	4	4	0.961	4	4	0.547	4	5	3	4	0.387	4	4	4	4	0.921
Insulin is more effective than oral hypoglycemic drugs?	2	1	1	1	1		0.345	4	4	0.413	3	4	0.000*	3	3	4	3	0.002*	4	4	4	4	0.033*
Do you think insulin administration is painful?	1	1	1	1	1		0.582	3	3	0.597	3	3	0.689	4	3	4	3	0.905	3	3	3	3	0.726
Insulin causes addiction	3	3	3	3	3		0.036*	2	2	0.000*	2	2	0.080	1.5	1	1	2	0.344	2	2	2	2	0.000*
Do you feel sympathy towards patients on insulin?	3	3	3	3	3		0.157	4	4	0.180	4	4	0.143	4	2	4	4	0.212	4	4	4	4	0.482
Do you believe that most patients on oral antidiabetic therapy do not accept insulin therapy?	1	1	1	1	1		0.156	4	4	0.994	4	4	0.906	4	4	4	4	0.405	4	4	4	4	0.000*
Do you believe that proper patient education and training are the keys to successful initiation of insulin therapy?	3	3	3	3	3		0.641	5	5	0.831	5	5	0.647	4	1	5	5	0.158	5	4	4.5	5	0.557
Do you think insulin compliance is difficult?	3	3	3	3	3		0.022*	3	3	0.374	3	3	0.486	3	5	4	3	0.07	3	3	4	3	0.329
Do you believe that the benefits of insulin therapy outweigh the risks of hypoglycemia and weight gain?	2	3	3	3	2		0.565	4	4	0.010*	4	4	0.152	4	2	4	4	0.345	4	4	4	4	0.005*

*The Kruskal-Wallis test is used to test the significance

The mean score for self-confidence was 25.37 ± 6.4, with a minimum score of 3 and a maximum score of 35. Only 13(2.8%) pharmacists had poor self-confidence, 63(13.5%) had good knowledge, while 341(73.3%) had excellent self-confidence. Table 11 shows the self-perceived confidence of pharmacists in counseling patients on insulin.

**Table 11:** Self-perceived confidence of pharmacists in counseling patients on insulin (*N* = 465)

Item	Very poor N (%)	Poor N (%)	Good N (%)	Very good N (%)	Excellent N (%)	Total N (%)
Ability to dispense insulin	62(14.9)	55(13.2)	131(31.5)	75(18.0)	93(22.4)	416(89.5)
Ability to counsel a patient on how to draw up the correct dose from the syringe	39(9.4)	43(10.3)	101(24.3)	100(24.0)	133(32.0)	416(89.5)
Ability to counsel a patient on proper injection technique	28(6.7)	34(8.2)	89(21.4)	97(23.4)	167(40.2)	415(89.2)
Ability to counsel a patient on proper insulin storage	21(5.0)	16(3.8)	67(16.1)	108(26.0)	204(49.0)	416(89.5)
Ability to counsel a patient on the proper timing of an insulin dose	40(9.7)	52(12.6)	100(24.2)	108(26.1)	114(27.5)	414(89)
Ability to counsel a patient on how to treat hypoglycemia caused by insulin	20(4.9)	41(10%)	113(27.4)	116(28.2)	122(29.6)	412(88.6)
Ability to counsel a patient on symptoms of hypoglycemia caused by insulin	21(5.1)	33(8)	87(21.2)	129(31.5)	140(34.1)	410(88.2)

Further analyses were conducted to investigate the influence of the pharmacists’ characteristics on their self-assessed confidence. Self-confidence scores differed statistically with sex (*P* = 0.018), where female patients’ scores were higher than males, years of experience (*P* = 0.000), and pharmacist’s direct exposure to diabetic patients (*P* = 0.007). However, the self-confidence scores of pharmacists were not influenced by age, university of graduation, or postgraduate studies. It is being found that self-confidence significantly increases as knowledge increases (Spearman’s rho = 0.367, *P* value = 0.000).

To ensure that the long period of the study did not affect the results of the questionnaire, a comprehensive analysis comparing data from two distinct cohorts: pre-COVID and post-COVID was conducted. Our findings revealed that the knowledge scores between the pre-COVID and post-COVID cohorts were remarkably consistent, with a minimal average difference of approximately 3% observed. Similarly, attitude scores across both cohorts showed negligible variance, with a difference of merely 0.3 points. Furthermore, an examination of socio-demographic characteristics and most other variables considered in our study demonstrated no significant differences between the cohorts. These results suggest that the temporal context of the COVID-19 pandemic had a limited effect on the primary outcomes of our research.

## Discussion

Insulin is frequently used in the management of both type 1 and type 2 diabetes mellitus. However, insufficient knowledge and malpractice on insulin self-administration could result in poor disease prognosis and insulin-related complications [13]. Pharmacists help control diabetes and enhance the quality of life by providing pharmaceutical care and prescription management services [25]. This is the first Egyptian cross-sectional study of insulin injection knowledge, attitude, and practice among pharmacists and patients in different healthcare settings across Cairo and Giza. The study uncovers certain areas of deficiencies in both pharmacists’ and patients’ knowledge. The study also reveals practice deficiencies among patients that can be addressed to improve their practices and thus diabetes care.

More than half of the patients in the current study were females, 75% of patients with a mean age of 52.4 ± 12.4. This could be because recruiting the patients was usually between 9 am and 2 pm in the hospital clinics so most of the patients were either female or older patients who were not working. This is quite similar to studies conducted in Egypt as 78.8% of patients were female with a median age of 52 years [26]. Most of the patients were using needles and syringes for insulin administration. This can be attributed to the fact that insulin syringes are provided free of charge through public hospitals, while pens should be paid for in some hospitals.

Although the American Diabetic Association has legislative recommendations for self-monitoring blood glucose levels using glucometer in all diabetic patients on insulin for better disease management and quality of life [27], 61.8% of our patients did not have access to glucometers despite being on insulin therapy for fairly long periods. This could be due to the low-income level among our patients and the inability to buy a glucometer. Also, it could be due to the illiteracy level among the patients and the inability to use the device. Other studies from developing countries like Kenya (59%) and Iraq (44%) also have reported low utilization of glucometers and adherence to self-monitoring [28, 29]. Given the importance of self-monitoring in improving glycemic control, there is a need to afford those low-income diabetic patients with glucometers and appropriate education on its use.

Regarding the level of knowledge among the study patients, only 2.6% of them had adequate knowledge, and 93.1% had moderately adequate knowledge. The overall knowledge percentage was 67.61%. This percentage was higher than the study conducted in India [30] and in line with a study conducted in Ethiopia [13] where the overall knowledge score was 46.9% and 63.4% respectively. The prime reason for good knowledge in the study patients may be because of better health facilities and accessibility in the study sites (Cairo and Giza). Regarding the pharmacists, knowledge assessment revealed that about 66.5% of the participants had moderately adequate knowledge, and only 59(12.7%) had adequate knowledge. The overall mean score among pharmacists was 61.2% which was higher than the overall mean score (49%) of pharmacists in the United Kingdom [31].

Our study found that female patients were more knowledgeable about insulin than males (*P* = 0.002). We also found that patients who have been on insulin treatment for a longer period are more likely to have better knowledge (*P* = 0.030). This could be attributed to the higher chances of exposure to information which helps the patients to obtain the knowledge.

Two main findings should be of concern both for pharmacists and patients. First, cloudy insulin must be mechanically resuspended so that it goes back into solution before injection [32]. Vigorous shaking should be avoided because this produces bubbles that will affect correct dosing [32]. However, in our study 38.2 and 12.2% of patients did it inappropriately (vigorous shaking) or did not know how to do this, respectively. These percentages were higher than in an Ethiopian study where 33.7% did not know that shaking insulin vials can make insulin more likely to clump [24]. Among the pharmacists, only (31.8%) of participants knew that insulin could be resuspended by either gentle mixing or rotating. Other pharmacists did not know the proper ways for resuspension. This poor knowledge may be due to inadequate training among pharmacists and not being up to date about current guidelines on diabetes management. Second, an interesting defect in knowledge was related to injecting insulin while it was cold, more than half (63.4%) of the patients did not know that injecting insulin while it was cold could increase the pain during injection. On the other hand, more than half (55%) of the pharmacists knew that insulin should not be injected while it’s cold. This could be translated as inadequate pharmacists’ counseling regarding the ways to minimize the pain during insulin injections.

Most of the patients (72.8%) had a positive attitude regarding insulin’s role in improving health and better controlling blood glucose, this was higher than studies conducted in Trinidad and Baghdad where only 43.6% and 34.5% thought that their glucose levels would be better controlled on insulin respectively [10, 33]. Regarding the pharmacists, half of them believed that diabetes is better controlled once insulin is started, so they can convince the patients who refuse to take insulin and those who believe that insulin has no privilege over oral hypoglycemic drugs.

Most of the patients and the pharmacists agreed that insulin therapy was more beneficial in regulating blood glucose levels in comparison with oral antidiabetic drugs; however, insulin injection was painful in 55.9% and 46.1% of the patients and pharmacists respectively. Most of them (60.4% of patients and 65.5% of pharmacists) did not agree with the fact that following the administration of insulin, a healthy diet was not needed to maintain blood glucose levels, same results were reported in a study conducted in Eastern India [34]. On the negative side, around half of the patients reported that they believe that regular use of insulin leads to addiction as they associate needles with drug abuse, and when they stop using insulin, they experience discomfort. This was similar to a Turkish study [35] and higher than a study conducted in Ethiopia [36], where half of the patients and only 7.3% reported that they believe that regular use of insulin leads to addiction respectively. On the other hand, only 14.5% of the pharmacists believed that insulin could cause addiction. So, pharmacists should educate patients that this is just a myth, and that insulin is a natural substance needed by the body and cannot get addicted to it.

Most of the patients (87.5%) felt self-conscious about taking injections in a public place while 10.6% felt that insulin administration is an embarrassment and 73.2% of pharmacists reported sympathy towards patients on insulin.

In the current study, 94% of the patients found that insulin therapy interfered with their daily activities and their social lives. This result might be a consequence of some factors. First, inadequate knowledge of appropriate storage conditions such as opened insulin vials could be stored at room temperature. Therefore, some patients when they go outside their home, they could skip their doses as they neither want to take nor have ice bags for insulin storage, and this could lead to insulin non-adherence. For that reason, 48.2% of the pharmacists agreed that insulin compliance is difficult as they thought that insulin therapy impacts lifestyle. Second, some of the patients (25.4%) believed that insulin is not easy to take and were dependent on others (pharmacists, family members, and neighbors) for injections, which may be a barrier to multiple daily insulin injections. Furthermore, the people who assist with injections may not always be available and this may lead to skipping insulin injections. A similar picture was observed in a Nigerian study, in which 28.2% did not inject themselves [37]. This finding further emphasizes the pharmacists’ role.

Although some aspects of practice were correctly followed by patients, all surveyed patients were making at least one insulin injection technique error. The three most common incorrect steps were skipping washing hands, not pressing on the skin for 5 seconds after pulling out the needle, and not checking for bleeding after injection. It is recommended to lift the skin as pinching up the skin decreases the chance of intramuscular injection [38], and most of the study subjects reported lifting the skinfold correctly. The most common correct practice observed in more than 89.2% of patients reported inserting the needle into the skin. This is an important step as insulin should be injected into the subcutaneous fat layer for better absorption while intradermal injections result in therapeutic ineffectiveness due to failure of delivering insulin at this site and increase the risk of local complications [39]. The ideal practice of keeping the needles under the skin for 5 seconds or longer after injecting was followed by 39.2% of the subjects in this study. Not leaving the needle after injection is found to be related to a higher frequency of insulin leakage from the site of injection [40].

Our study has several strengths. Our study patients were recruited from different hospitals located in Cairo and Giza, the two largest cities in Egypt that include different sociodemographic levels of the patients. The study ensured good representation of pharmacists as it included pharmacists from different sectors in Cairo and Giza districts including districts with different socioeconomic levels and distant agricultural and rural districts.

Despite these strengths, our study has several limitations. One study limitation is that the researcher almost visited the pharmacies in the time between 9 am and 9 pm. No data was obtained from the 9 pm to 9 am shift. Replication of the study in different parts of Egypt is recommended to test the generalizability of our findings. Another limitation of the study is that some pharmacists asked to finish the survey in a few days to complete it in their spare time which could affect the data collected in the knowledge section. The study has additional limitations, such as no random selection for patients, cross-sectional design, and social desirability bias for attitude.

This study recommends improving patients’ knowledge about diabetes management in several ways as continuous audio and video display when the patients are waiting in the clinics, a flyer with illustrations could be given to the patient that contains information on different types of insulin with their color code, sites of insulin administration, techniques of insulin administration, storage of insulin, signs of hypoglycemia and hyperglycemia, complications of insulin and its management [41].

## Conclusion

This study identifies considerable gaps in patients’ and pharmacists’ knowledge about insulin therapy. The vital part of comprehensive diabetes care is the information and education which leads to improvement in knowledge, attitude, and practice. The findings of this study could be useful for policy or decision makers, healthcare providers, and patient support groups who may need to design interventions to improve the health outcomes of patients with diabetes. This might help the patients to have a better understanding of the self-administration of insulin and improve their practice skills. This study also strongly recommends higher education and a more structured pharmacist training schedule. It also shows a willingness among most pharmacists to attend workshops dealing with insulin therapy. Different learning strategies may be of interest to pharmacists who regularly assist diabetic patients but cannot attend such workshops.

### Supplementary Information

**Supplementary Material 1.**

**Supplementary Material 2.**

## Data Availability

The datasets generated during and/or analyzed during the current study are available from the corresponding author upon reasonable request.

## Abbreviations

DM: Diabetes mellitus
KAP: Knowledge, attitude, and practice
SPSS: Statistical packages for social science
N: Number
SC: Subcutaneous
DF: Degree of freedom
