# Supplementary material for: Knowledge, attitude, and practice of insulin among diabetic patients and pharmacists in Egypt: “cross-sectional observational study”
Source: BMC Med Educ. 2024 Apr 9;24:390. doi: 10.1186/s12909-024-05367-5 (PMC11005131; doi:10.1186/s12909-024-05367-5)
Supplement: Supplementary file 1 — Supplementary Material 1. [file 12909_2024_5367_MOESM1_ESM.docx]

**Patient Questionnaire**

**Serial number: …**

**Date: ……… start time: … end time: …**

*Dear patient,*

This survey is part of a study conducted, to assess patient's knowledge, attitude, and practice regarding insulin, in the faculty of Pharmacy Cairo University under the title of: ***"Knowledge, attitude, and practice towards insulin among diabetic patients and pharmacists in Egypt"***

By filling out the provided questionnaire, you agree to participate in this study. All data will be kept confidential and your cooperation is highly appreciated.

We would be grateful if you participate in this study

**Part 1: demographics**

| 1. Sex:   Male Female  c  c | 1. Age: |
| --- | --- |
| 1. Education:   Primary Secondary  c  c  Higher education Not educated  c  c | 1. Employment:   Unemployed Self-employed  c  c  Government employed Retired  c  c |
| 1. Residence:   Urban area Rural area  c  c | |
| 1. Do you check your blood glucose at your home using a glucometer? Yes No   c  c  If yes, who trained you:  Doctor  c  Pharmacist  c  Nurse  c  Other, please specify  c | |
| 1. Method of insulin administration   Pen Needle/syringe Pump  c  c  c | 1. Duration of insulin use   <6 months 6-12months  c  c  >1year-10 years > 10 years, please specify  c  c |

**Part 2: knowledge, attitude, and practice**

1. knowledge about insulin

| 1. How should short-acting insulin be taken concerning meals?   With meals At any time  c  c  Before meals Do not know  c  c | 1. Insulin should be injected:   Subcutaneously  c  Intramuscular  c  Do not know  c | | |
| --- | --- | --- | --- |
| 1. If two types of insulin are to be mixed, is/are there any precaution(s) you should take concerning the vial?   Shaking 2 Gentle mixing Rotating  c  c  c  Gentle mixing or rotating Do not know  c  c | 1. What are the sites of insulin injections?   ****One or multiple answers***  c  Abdomen  Thigh  c  Upper arm  c  Do not know  c | | |
| 1. for minimizing pain associated with insulin injections: | True | False | Do not know |
| 1. Injecting insulin while it is cold |  |  |  |
| 1. Using a thick needle |  |  |  |
| 1. Removing air bubbles from the insulin syringe before injecting |  |  |  |
| 1. The angle to administer insulin injection is 45 degrees.   Yes No Do not know  c  c  c | 1. You should rotate the injection site.   c  c  Yes No Do not know  c | | |
| 1. Insulin should be stored in | | | |
| 1. The syringe should be used for one time only.   Yes No Do not know  c  c  c | 1. Do you know the side effect/s of insulin?   Yes No  c  c   1. If yes, please mention | | |

1. Attitude towards insulin

| **Item** | **1-Disagree** | **2-Neutral** | **3-Agree** |
| --- | --- | --- | --- |
| 1. Do you think insulin administration with a pen is easier than syringes? |  |  |  |
| 1. Insulin started due to worsened DM |  |  |  |
| 1. Do you think your DM has been better controlled since you started insulin? |  |  |  |
| 1. Insulin makes life less flexible |  |  |  |
| 1. Insulin allows a less restrictive diet |  |  |  |
| 1. Insulin therapy is costly |  |  |  |
| 1. Insulin is more effective than oral hypoglycemic drugs |  |  |  |
| 1. Do you think insulin administration is time-consuming? |  |  |  |
| 1. Do you think insulin administration is an embarrassment? |  |  |  |
| 1. Do you think insulin is easy to take? |  |  |  |
| 1. Do you think insulin is painful? |  |  |  |
| 1. Are you fearful of weight gain resulting from insulin? |  |  |  |
| 1. Are you afraid of scarring at the injection sites caused by insulin injections? |  |  |  |
| 1. Insulin causes addiction |  |  |  |
| 1. Does insulin interfere with your daily activity? |  |  |  |

1. Steps of insulin injection:

| Item | 1-Yes | 2-No |
| --- | --- | --- |
| - Wash your hands well. |  |  |
| - Gently pinch a two- to three-inch fold of skin on either side of the cleaned injection site. |  |  |
| - Insert the needle into the skin. |  |  |
| - Leave the skin and leave the syringe in place for 5 seconds after injecting. |  |  |
| - Pull the needle out and press on the skin for 5 seconds. |  |  |
| - Check for bleeding. |  |  |

**Part 4: Satisfaction of patients with physician-patient interaction**

1. Are you satisfied with your physician?

Yes No

c

c

1. If no, why?

Not enough time for consultation No follow-up

c

c

1. If yes, please rate your Patient-Physician interaction.

| **Experience of patients with Physician** | **Poor** | **Good** | **Excellent** |
| --- | --- | --- | --- |
| Feel free to talk to your physician |  |  |  |
| Enough time for consultation |  |  |  |
| Ease of contacting your physician |  |  |  |

**Thank you for taking time to complete this questionnaire. Your input is important. All information you provide is confidential & will only be used for research purposes.**
