# Supplementary material for: Knowledge, attitude, and practice of insulin among diabetic patients and pharmacists in Egypt: “cross-sectional observational study”
Source: BMC Med Educ. 2024 Apr 9;24:390. doi: 10.1186/s12909-024-05367-5 (PMC11005131; doi:10.1186/s12909-024-05367-5)
Supplement: Supplementary file 2 — Supplementary Material 2. [file 12909_2024_5367_MOESM2_ESM.docx]

**Pharmacist questionnaire**

**Serial number: …**

**date: … start time: … end time: …**

*Dear pharmacist,*

This survey is part of a study conducted, to assess pharmacist's knowledge and attitude regarding insulin, in the faculty of Pharmacy Cairo University under the title of: ***"Knowledge, attitude, and practice towards insulin among diabetic patients and pharmacists in Egypt"***

By filling out the provided questionnaire, you agree to participate in this study. All data will be kept confidential and your cooperation is highly appreciated.

e would be grateful if you participate in this study

**Part 1: demographics**

| 1. Sex:   Male Female  c  c | 1. Age: |
| --- | --- |
| 1. Year of graduation: |  |
| 1. Type of graduating school or university: | |
| 1. Postgraduate studies: 1 Yes 2 No   If yes,  MSc Ph.D. 3 Pharm D Diploma  c  c  c  c | 1. Practicing site: |
| 1. Years of practice as a pharmacist:   <1 year 1-5 years 6-10 years>10 years  c  c  c  c | 1. Do you have direct exposure to patients on insulin?   Yes No  c  c |

**Part 2: knowledge and attitude towards insulin**

Section I (Knowledge)

| 1. Insulin is:  - Hormone - Enzyme - Do not know | 1. Insulin is secreted by the pancreas in:  - Alpha cell - Beta cell - Delta cell - Do not know | | |
| --- | --- | --- | --- |
| 1. How comfortable do you feel when managing a diabetic patient?  - Very comfortable - Somewhat comfortable - Somewhat uncomfortable - Very uncomfortable | 1. Which patient requires insulin?  - Type 1 DM - Type 2 DM - Both - Do not know | | |
| 1. What are the sites of insulin injections?   ****one or multiple answers***   - Abdomen - Gluteus - Deltoid - Thigh - Do not know | 1. The distance to rotate on the same site is one thumb:  - True - False - Do not know | | |
| 1. Unopened insulin vials should be stored in:   ***one or multiple answers**   - Room temperature - Refrigerator - Freezer - Do not know | 1. Used insulin vials should be stored in:   ***one or multiple answers**   - Room temperature - Refrigerator - Freezer - Do not know | | |
| 1. If a fridge is not available insulin vial could be stored in a clay pot containing water:   c  True False Do not know  c  c | 1. The insulin pen has a needle of 31 gauge.   c  True False Do not know  c  c | | |
| 1. Women who get pregnant should stop insulin.   True False Do not know  c  c  c | 1. How should short-acting insulin be taken concerning meals?   With meals At any time  c  c  Immediately before or directly after Do not know  c  c | | |
| 1. for minimizing pain associated with insulin injections: | True | False | Do not know |
| 1. injecting insulin while it is cold |  |  |  |
| 1. Using a 29 gauge needle |  |  |  |
| 1. Removing air bubbles from the insulin syringe before injecting |  |  |  |
| 1. IF two types of insulin are to be mixed, is/are there any precaution(s) you should take concerning the vial?   Shaking Gentle mixing Rotating  c  c  c  Gentle mixing or rotating Do not know  c  c | 1. Which of the following is/are side effect/s of insulin?   ***One or multiple answers**  Weight gain Hypoglycemia Allergy  c  c  c    Erectile dysfunction Do not know  c | | |
| 1. Diabetic ketoacidosis can be developed in   Type 1 DM Type 2DM  c  c  Both Do not know  c  c | 1. Hypoglycemia is blood glucose level….   <50mg/dl <70mg/dl  c  c  c  <80mg/dl <100mg/dl Do not know  c  c  c  c | | |
| 1. What are the signs of hypoglycemia?   Sweating  c  Anxiety  c  Blurred vision  c  Tingling lips  c  None of the above  c  All of the above  c | | | |
| 1. What are the contraindications for insulin? | | | |

1. knowledge regarding preparation for insulin injection

| Item | 1-True | 2-False | 3-Do not know |
| --- | --- | --- | --- |
| 1. The hands should be washed with soap and water before handling injection devices |  |  |  |
| 1. The insulin vial should be kept at room temperature at least for 15 minutes before giving an injection |  |  |  |
| 1. Air should be injected into the insulin vial before drawing insulin out of the vial |  |  |  |
| 1. If drawing both soluble and isophane insulin into the same syringe the isophane insulin should be drawn into the vial first |  |  |  |

1. Which of the following is/are the most preferred method for learning about DM and insulin? ***One or multiple answers**

- Online courses
- Workshops
- Publications distributed to the pharmacy
- Another method, please mention it
- Not interested

Section II (Attitude)**: **only one answer is required***

| **Item** | **1-Strongly disagree** | **2-Disagree** | **3-Neutral** | **4-Agree** | **5-Strongly agree** |
| --- | --- | --- | --- | --- | --- |
| 1. Do you think DM is better controlled once insulin is started? |  |  |  |  |  |
| 1. Do you think Insulin allows a less restrictive diet? |  |  |  |  |  |
| 1. Insulin therapy is costly? |  |  |  |  |  |
| 1. Insulin is more effective than oral hypoglycemic drugs? |  |  |  |  |  |
| 1. Do you think insulin administration is painful? |  |  |  |  |  |
| 1. Insulin causes addiction |  |  |  |  |  |
| 1. Do you feel sympathy towards patients on insulin? |  |  |  |  |  |
| 1. Do you believe that most patients on oral diabetes therapy do not accept insulin therapy? |  |  |  |  |  |
| 1. Do you believe that proper patient education and training are the keys to the successful initiation of insulin therapy? |  |  |  |  |  |
| 1. Do you think insulin compliance is difficult? |  |  |  |  |  |
| 1. Do you believe that the benefits of insulin therapy outweigh the risks of hypoglycemia and weight gain? |  |  |  |  |  |

**Part 3: Pharmacist perceptions of confidence in the ability to counsel on the safe use of insulin:**

| Question | 1 | 2 | 3 | 4 | 5 |
| --- | --- | --- | --- | --- | --- |
| 1. Ability to **dispense** insulin |  |  |  |  |  |
| 1. Ability to counsel a patient on how to **draw up the correct** dose from the syringe |  |  |  |  |  |
| 1. Ability to counsel a patient on proper **injection technique** |  |  |  |  |  |
| 1. Ability to counsel a patient on proper insulin **storage** |  |  |  |  |  |
| 1. Ability to counsel a patient on the proper **timing** of an insulin dose |  |  |  |  |  |
| 1. Ability to counsel a patient on how to **treat** hypoglycemia caused by insulin |  |  |  |  |  |
| 1. Ability to counsel a patient on **symptoms of hypoglycemia** caused by insulin. |  |  |  |  |  |

*On a scale of 1 – 5 with 1 being “poor” and 5 being “excellent,” how would you rate your confidence when counseling a patient on insulin?

**Thank you for taking time to complete this questionnaire. Your input is important. All information you provide is confidential & will only be used for research purposes.**
